# Routes into Single Motherhood, Normative Family Beliefs, and Perception-Based Indicators of Psychosocial Vulnerability Among Women Recruited Through Ecuadorian Public University Networks: An Exploratory Study

**DOI:** 10.3390/bs16101798

**Published:** 2026-10-02

**Authors:** Holguer Estuardo Romero-Urrea, Guillermo Fernando León-Samaniego, Carmen Graciela Zambrano Villalba, Dolores Guadalupe Saldarriaga Jiménez, Gabriel José Suárez Lima, Patricio Alvarez-Muñoz, Marco Faytong-Haro

**Affiliations:** 1 Facultad de Salud y Servicios Sociales, Universidad Estatal de Milagro, Milagro 091050, Ecuador; gleons1@unemi.edu.ec (G.F.L.-S.); czambranov@unemi.edu.ec (C.G.Z.V.); dsaldarriagaj@unemi.edu.ec (D.G.S.J.); gsuarezl@unemi.edu.ec (G.J.S.L.); 2 Facultad de Ciencias Sociales, Educación Comercial y Derecho, Universidad Estatal de Milagro, Milagro 091050, Ecuador; palvarezm@unemi.edu.ec; 3 Facultad de Economía, Universidad Estatal de Guayaquil, Guayaquil 090506, Ecuador; 4 Facultad de Investigación, Universidad Estatal de Milagro, Milagro 091050, Ecuador; mfaytongh@unemi.edu.ec

**Keywords:** single motherhood, anticipated stigma, self-esteem, normative family beliefs, motherhood penalty, work–family conflict, Ecuador

## Abstract

Single motherhood may begin through a self-reported decision to raise a child alone or through circumstances such as paternal irresponsibility, partner abandonment, and family constraints. Guided by the stress-process model and perspectives on stigma and self-determination, this exploratory cross-sectional study examined associations among the reported route into single motherhood, normative beliefs about the indispensability of fathers, perceived labor inequity, and two perception-based indicators: endorsement of fear of judgment and endorsement of changes in self-esteem/self-concept. Participants were recruited through purposive networks coordinated by one faculty or administrative contact at each of the six public universities in Coastal and Andean Ecuador. The resulting non-probability analytic sample comprised 157 women who provided consent and identified as single mothers. Of these respondents, 51 (32.5%) selected the decision-based response (“she decided to do it alone”), and 106 (67.5%) selected one of the three circumstance-based responses. Fear of judgment was endorsed less often in the decision-based group than in the circumstance-based group (9.8% vs. 25.5%; exploratory *p* = 0.022). In secondary exploratory logistic regressions, both membership in the decision-based group (aOR = 0.25, 95% CI 0.08–0.77) and student status rather than faculty status (aOR = 0.18, 95% CI 0.06–0.55) were associated with lower within-sample odds of fear-of-judgment endorsement. The reported route was not independently associated with changes in self-esteem/self-concept. This indicator was associated with considering a father indispensable (aOR = 2.91, 95% CI 1.41–5.99) and with greater perceived inequity in labor (aOR = 1.57 per index point, 95% CI 1.15–2.13). Among these respondents, the selected indicators varied according to reported route, normative beliefs, and perceived labor inequity. Because the sample was non-probability-based and the outcomes were derived from individual questionnaire responses, the findings are not population estimates. They identified questions for larger studies using stronger sampling designs and validated multidimensional measures, as well as potential areas for university assessment, including stigma, caregiving accommodations, and employment equity.

## 1. Introduction

### 1.1. Stress Process and Heterogeneity in Single Motherhood

Single motherhood is not a uniform psychosocial experience. The stress-process model helps to explain this variation. It links social position to three related elements: exposure to discrete events and chronic strains, access to coping and social resources, and outcomes such as distress or changes in self-concept (Pearlin et al., 1981). Within this framework, single motherhood is not inherently distressful. Any observed disadvantage may reflect greater exposure to stressors, fewer protective resources, or both. Comparative studies often report greater psychological distress among single mothers than among partnered mothers, while studies focused on single mothers associate financial hardship, social disadvantage or isolation, chronic stress, and limited support with poorer mental health or quality of life (Cairney et al., 2003; Crosier et al., 2007; G. E. Kim et al., 2018; G. E. Kim & Kim, 2020; Rousou et al., 2016, 2019; Targosz et al., 2003). Social support may help to buffer these pressures (Cohen & Wills, 1985). Accordingly, the present study considers the reported route to single motherhood alongside the surrounding social conditions, without assuming that family structure alone causes psychosocial vulnerability.

Research on planned solo motherhood illustrates why the routes to single motherhood should not be treated as equivalent. A recent systematic review examined the motivations for solo motherhood and experiences with assisted reproduction (Zamora-Martínez et al., 2025). One study described a strong desire for parenthood, active choice, and early parenting experiences (Murray & Golombok, 2005), while another examined how solo mothers represent sperm donors within their family narratives (Zadeh et al., 2016). Two studies comparing planned solo mother families with partnered families found broadly similar mother–child relationships and no significant disadvantage in child psychological adjustment (Golombok et al., 2016, 2021). These findings, together with research on parental gender, caution against treating a resident father as universally essential (Biblarz & Stacey, 2010; Silverstein & Auerbach, 1999). However, this does not mean that the absence of fathers has no consequences. Studies using stronger designs continue to identify adverse effects on selected child outcomes, although these effects are generally smaller than those estimated from conventional cross-sectional studies (McLanahan et al., 2013). The review also notes that estimates must be interpreted alongside selection and related family and socioeconomic conditions of the children. Planned donor-conception samples are more narrowly defined than the decision-based category used in this study. In this study, decision-based refers only to respondents who selected “she decided to do it alone.” The response does not indicate when the decision was made and is not a direct measure of planned single motherhood, reproductive autonomy, or agency.

### 1.2. Stigma and Normative Family Beliefs

Although family structure does not inherently produce distress, the social devaluation of a family form may affect behavior and well-being. Stigma develops through labeling, stereotyping, separation, status loss, and discrimination within unequal power relations (Link & Phelan, 1995). Whether people anticipate evaluation, experience identity threat, or use particular coping responses depends partly on how salient a stigmatized identity becomes in a given context (Frost, 2011; Major & O’Brien, 2005; Stuber et al., 2008). Stigma may then affect individual and population health through chronic stress, restricted access to resources and institutional exclusion (Frost, 2011; Hatzenbuehler et al., 2013; Stuber et al., 2008). Among divorced single-parent women, self-stigma was associated with poorer mental health, and self-esteem mediated this association (A. Kim et al., 2023). This finding is relevant to the present focus on self-evaluation, although it does not establish a temporal order.

Normative beliefs about motherhood may intensify these effects. The ideal of intensive motherhood portrays good mothering as child-centered, expert-guided, emotionally absorbing, and personally sacrificial (Arendell, 2000; Hays, 1996). Studies have associated intensive or idealized mothering with stress, poorer psychological well-being, parental burnout, and reduced work–family balance (Henderson et al., 2016; Meeussen & Van Laar, 2018; Rizzo et al., 2013). Maternal guilt and shame are also associated with self-discrepancy and fear of negative evaluation (Liss et al., 2013). For a woman parenting without a partner, believing that a father is indispensable may create a standard against which she evaluates her family. This interpretation is hypothetical because the study did not measure internalized stigma.

### 1.3. Employment Inequality, Work–Family Conflict, and the University Setting

Normative expectations also extend to paid employment. Employment research documents motherhood-related penalties in hiring, competence evaluations, wages and career progression (Benard & Correll, 2010; Budig & England, 2001; Correll et al., 2007; Cuddy et al., 2004; Cukrowska-Torzewska & Matysiak, 2020; Deming, 2022; Gangl & Ziefle, 2009). These disadvantages may reinforce the perception that caregiving and full participation in paid work are incompatible for women. They may also coexist with work–family conflict, which arises when time, strain, or behavioral demands make pressures from work and family roles incompatible (Greenhaus & Beutell, 1985). Among working mothers, recent correlational research has associated better work–family balance and greater satisfaction of basic psychological needs with better emotional well-being (Olivieri et al., 2024). More broadly, work–family conflict is associated with a range of work, family, and health outcomes (Amstad et al., 2011), whereas workplace social support is associated with lower work–family conflict (Kossek et al., 2011).

Universities are particularly relevant because women may occupy overlapping roles as students, employees, and caregivers. A qualitative study of Iranian student mothers describes the difficulties in combining motherhood with the student role (Behboodi Moghadam et al., 2017). In Ecuador, a narrative review describes the broader gendered institutional disadvantage among poor rural single mothers in the highlands (Pino Gavidia et al., 2022). Research in other university settings reports difficulties balancing study and caregiving, time constraints, disrupted academic participation, and limited institutional support among student-parents (Evans, 2025; Moreau & Kerner, 2015). A study at the Universidad Nacional de Chimborazo reported delayed completion and graduation among some single student mothers (Cargua et al., 2024). National census data provide a broader context: in 2022, 4,624,351 women living in Ecuador were mothers, and 1.7 million were listed as household representatives (INEC, 2024). These figures do not identify single mothers or their university affiliations. Evidence outside universities further illustrates how eligibility rules may disadvantage single-parent families: A Spanish commentary described one case in which school-meal grant requirements disadvantaged single-parent families and were interpreted by the authors as gender-based discrimination (Obón Azuara et al., 2023).

Therefore, the university context brings caregiving into contact with both education and employment. Students and faculty members were included because they occupied these two institutional positions. The university role was included in the adjusted models, but the groups may also differ in socioeconomic circumstances, occupational demands, and exposure to evaluation that were not fully measured. Their inclusion permits exploratory comparison; however, it does not imply that the groups are otherwise comparable.

### 1.4. Conceptual Framework and Study Objectives

The studies reviewed above do not directly examine the relationships considered here among women affiliated with Latin American universities in the same way. This study examines self-reported routes into single motherhood in relation to anticipated judgment, self-evaluation, fatherhood beliefs, and perceived labor inequity. The analysis is organized using stress-process and stigma perspectives. The two outcomes are treated as perception-based indicators: fear-of-judgment endorsement relates to anticipated stigma, and self-esteem/self-concept-change endorsement relates to perceived self-evaluation. Belief in father indispensability represents a normative family belief, whereas perceived labor inequity represents potential institutional strain. This study also draws on self-determination theory, which identifies autonomy, competence, and relatedness as basic psychological needs (Ryan & Deci, 2000). Because autonomy and agency were not measured, the theory was used only to frame future questions and not to interpret the decision-based category as a measure of autonomy.

Within the recruited group, the primary objective was to compare the endorsement of fear of judgment and changes in self-esteem/self-concept across the reported-route groups. The secondary objective was to examine whether belief in the indispensability of fathers and perceived labor inequity were associated with the endorsement of changes in self-esteem/self-concept. We expected that fear-of-judgment endorsement would be less common in the decision-based group. We also expected that the endorsement of changes in self-esteem/self-concept would be positively associated with father-indispensability beliefs and perceived labor inequity. All comparisons and adjusted models were treated as exploratory within-sample associations and not as causal or population-level effects.

## 2. Materials and Methods

### 2.1. Study Design and Setting

This exploratory cross-sectional study surveyed women from six public universities in the Coastal and Andean regions of Ecuador. Data were collected from 2 June to 28 July 2023. Participants completed a structured online questionnaire in Spanish using Google Forms, and the analysis included only quantitative responses submitted during those dates. As predictors and outcomes were measured simultaneously, the study could not establish temporal order or causal direction.

### 2.2. Recruitment and Participants

Recruitment followed a purposive, network-based strategy because the participating institutions did not maintain research-accessible registries of single mothers. At each university, a faculty or administrative contact familiar with the student and employee networks circulated the survey invitation. The contact was shared primarily with women in those networks who were known to be single mothers, and recipients could forward it to peers. Potential participants accessed the questionnaire directly through an online link. The central research team did not receive any names or personal contact information.

This strategy made it possible to reach a population that was not readily identifiable in institutional records; however, it did not produce a probability sample. Women with stronger social ties, greater institutional visibility or engagement, closer connections to university contacts, or greater willingness to discuss their experiences may have been more likely to participate in the study. However, the direction and magnitude of this selection cannot be determined. Therefore, the sample does not represent all single mothers at the participating universities or across Ecuadorian public universities.

The questionnaire received 188 submissions between 2 June and 28 July 2023. Respondents were included in the analysis if they provided informed consent, identified as female, and identified themselves as single mothers. Two respondents did not consent, seven did not identify as female (four identified as male, and three preferred not to answer), and 22 did not identify as single mothers. The final analytical sample consisted of 157 women.

Because the invitation circulated through targeted networks, no sampling frame was available, and the number of women who received the link was not recorded in the survey. Therefore, the response rate could not be calculated. The estimates describe only the 157 respondents reached through these networks and included in the analysis.

### 2.3. Questionnaire, Classification of the Route into Single Motherhood, and Measures

The research team used the 34-item Cuestionario Estado Emocional de las Madres Solteras (Emotional State of Single Mothers Questionnaire), a Spanish-language instrument covering reasons for single motherhood, perceived emotional changes, family and social positioning, religious framing, fatherhood beliefs, and work-related perceptions. The questionnaire content was evaluated in a separate, published instrument-development study (Zambrano Villalva et al., 2023). Content validation was not conducted in the present analysis. In a separate study, 12 experts assessed the relevance, coherence, sufficiency, and clarity of all 34 items. After revision in response to expert feedback, the authors reported an interexpert agreement of kappa = 0.731 (*p* < 0.001) and a content validity index of 1.00. The present analysis used a questionnaire to examine selected item responses. The expert review provided evidence for the content validity of the questionnaire as a whole. However, it does not establish that the individual response options used here are validated psychometric measures, and it provides no evidence of their construct validity, dimensionality, or reliability.

Several questions allowed for more than one response. For example, one item asked about the emotions associated with single motherhood when a child reaches adolescence and included the option “fear of being judged for her decision.” Another question asked about emotional changes during the first months of single motherhood and included “self-esteem and self-concept” as an option. Therefore, the analysis captures the endorsement of selected perceptions rather than diagnoses, symptom scales, or psychometric measures of stigma, fear, self-esteem, or self-concept.

The reported route to single motherhood was classified based on the item asking why the respondent was a single mother. Women who selected “she decided to do it alone” were assigned to the decision-based group. Those who selected paternal irresponsibility, partner abandonment, or the absence of another alternative because of family circumstances were assigned to the circumstance-based group.

These categories describe only the selected questionnaire responses. The decision-based group may include individuals with varied relationship histories and circumstances. The response does not indicate whether the decision preceded conception, whether assisted reproduction was used, or whether the respondent experienced greater autonomy or agency. Therefore, it is not equivalent to the narrower concept of planned single motherhood by choice. Current marital status was not used in this classification because a woman may have entered single motherhood without a partner and later married or entered a different relationship.

Two binary perception-based indicators were used as primary outcomes. Fear-of-judgment endorsement was coded when a respondent selected “fear of being judged for her decision” in the multiple-response item concerning emotions when a child reaches adolescence. Self-esteem/self-concept-change endorsement was coded when a respondent selected “self-esteem and self-concept” in the item concerning emotional changes during the first months of single motherhood. These labels are shorthand for endorsement of specific response options and do not represent the multidimensional constructs assessed by validated stigma or self-esteem scales.

Secondary indicators included pride in child achievements; mixed feelings; negative social positioning; negative religious framing; belief that a father is indispensable; perceived inequality in job access, pay, and labor rights; perceived changes in workplace treatment; and perceived deficient work performance.

Negative social positioning included every response other than “accepted” to the item asking how single mothers are regarded socially. Negative religious framing included responses referring to disrupted family harmony, disorientation or insecurity, or impaired spiritual balance; the supportive response “need to be comforted and supported” served as the reference. The perceived labor inequity index summed three binary items on unequal job access, remuneration, and labor rights, producing scores ranging from 0 to 3. The internal consistency was moderate (Cronbach’s alpha = 0.693). Age was analyzed in five-year increments, and university role was classified as either student or faculty. The questionnaire did not record concurrent employment among students, the working conditions of employed students, or the faculty rank and seniority.

### 2.4. Statistical Analysis

The primary analysis was descriptive in nature. Participant characteristics and survey indicators were summarized using means, standard deviations, medians, ranges, frequencies, and percentages. Decision-based and circumstance-based groups were compared exploratorily using Pearson chi-square tests without continuity correction for categorical indicators and Welch’s t-tests for age and the labor inequity index.

Two multivariable binary logistic regressions were used as secondary exploratory analyses of within-sample associations. The first model examined fear-of-judgment endorsement using route group, age, and university role. The second model of self-esteem/self-concept-change endorsement used route group, age, university role, belief that a father is indispensable, and the perceived labor inequity index. The predictors were selected a priori and were conceptually distinct. The first model included 32 outcome events for three predictors (10.7 events per predictor), and the second model included 62 events for five predictors (12.4 events per predictor) (Vittinghoff & McCulloch, 2007).

Both models used the HC0 sandwich covariance estimates (White, 1980; Zeileis, 2006). These estimates are robust to some forms of variance misspecification, but they cannot correct for the selection bias introduced by non-probability recruitment. Multicollinearity was assessed using variance inflation factors. Model adequacy was summarized using likelihood ratio tests, McFadden pseudo-R-squared, Hosmer–Lemeshow tests based on 10 risk groups, apparent c-statistics, Brier scores, leverage, and Cook distance. These diagnostics evaluate the fitted models within the analytic sample and do not constitute external validation.

The results are reported as adjusted odds ratios (aORs) with 95% confidence intervals (CIs). Two-sided *p*-values below 0.05 were treated as exploratory indicators rather than definitive evidence. One age reported in words was recoded, after which no model variables had missing data. Analyses were conducted using R version 4.4.3 (R Foundation for Statistical Computing, Vienna, Austria). Base R was used for descriptive statistics, chi-square tests, Welch’s t-tests, and logistic regressions; the sandwich and lmtest packages were used for the HC0 covariance matrices and robust coefficient tests. Site-level comparisons and cluster-adjusted models were not conducted because university affiliation was recorded inconsistently in free-text fields, and only six recruitment sites participated.

### 2.5. Ethical Considerations

The study was conducted in accordance with the Declaration of Helsinki (World Medical Association, 2013) and approved by the Ethics Committee of the Technical University of Manabí (CEISH-UTM; protocol CEISH-UTM-EXT_23-1-18_HERU; 31 May 2023). The first page of the online questionnaire presented the study information and an informed consent question. Only respondents who provided consent were included in the study. Participation was voluntary, and the analytical dataset did not include direct personal identifiers.

## 3. Results

### 3.1. Participant Characteristics and Reported Route into Single Motherhood

The 157 participants had a mean age of 29.99 years (SD = 10.04), a median age of 27 years, and an age range of 18–74 years. Most were students (n = 132, 84.1%), and the remaining 25 (15.9%) were faculty members. The questionnaire did not collect information on employment status among students or academic rank and seniority among faculty.

Fifty-one women (32.5%) selected the decision-based response, “she decided to do it alone.” The other 106 women (67.5%) reported circumstance-based reasons: paternal irresponsibility (n = 77), partner abandonment (n = 27), or family constraints (n = 2). In exploratory comparisons, age and university role did not differ significantly between the groups (Table 1). At the time of the survey, 132 participants were single, 16 were divorced, seven were married, one was widowed, and one was separated. These current statuses were not used to classify the route into single motherhood, which refers to how the experience began.

### 3.2. Emotional, Social, and Labor Indicators

Fear of judgment was endorsed by 32 participants (20.4%). The proportion was lower in the decision-based group than in the circumstance-based group (9.8% vs. 25.5%, *p* = 0.022). Self-esteem/self-concept change was endorsed by 62 participants (39.5%); the difference between the two route groups was not statistically significant (31.4% vs. 43.4%, *p* = 0.149). Pride in a child’s achievements was the most common emotional endorsement (74.5%) and did not differ between the groups. Negative social positioning was endorsed by 72.6% of the participants, while 43.3% selected negative religious framing. Approximately one-third of the respondents considered the father indispensable (33.1%). Perceived workplace disadvantages were also common: 33.1% reported unequal job access, 38.9% unequal pay, and 30.6% unequal labor rights. Most participants (86.0%) indicated that workplace treatment changed at some stage of pregnancy, childbirth, early childhood, or consistently (Table 2).

### 3.3. Secondary Exploratory Multivariable Associations

In the first secondary model, the decision-based group had lower within-sample odds of fear-of-judgment endorsement than the circumstance-based group after adjusting for age and university role (aOR = 0.25, 95% CI 0.08–0.77, *p* = 0.016). Students also had lower odds of endorsement than faculty members (aOR = 0.18, 95% CI 0.06–0.55, *p* = 0.003). Age was not independently associated with the outcomes. These estimates describe only the recruited respondents and are not representative of the population parameters.

In the second model, the reported route was not independently associated with self-esteem/self-concept-change endorsement (decision-based vs. circumstance-based: aOR = 0.64, 95% CI 0.30–1.36, *p* = 0.247). Two other variables were associated with this outcome in the recruited sample. Participants who considered a father indispensable had higher odds of endorsement (aOR = 2.91, 95% CI 1.41–5.99, *p* = 0.004), as did participants with higher scores on the labor inequity index (aOR = 1.57 per one-point increase, 95% CI 1.15–2.13, *p* = 0.004). Age and university role did not meet the exploratory significance threshold in this model (Table 3). The estimates do not establish a temporal direction or causal effects.

Both models converged and exhibited minimal multicollinearity. The VIFs ranged from 1.01 to 1.20 in the fear-of-judgment model and from 1.02 to 1.23 in the self-esteem/self-concept-change model. Overall, the likelihood-ratio tests were significant for both the fear-of-judgment model (chi-square = 14.69, df = 3, *p* = 0.002; McFadden pseudo-R-squared = 0.092) and the self-esteem/self-concept-change model (chi-square = 22.10, df = 5, *p* < 0.001; McFadden pseudo-R-squared = 0.105).

Hosmer–Lemeshow tests did not indicate a gross lack of fit (chi-square = 6.78, df = 8, *p* = 0.560; and chi-square = 8.01, df = 8, *p* = 0.433, respectively). The apparent c-statistics were 0.668 and 0.713, and the Brier scores were 0.145 and 0.208, respectively. The maximum Cook’s distances were 0.088 and 0.069, both below 1. These results indicate that the models were numerically adequate for the analytic sample. However, they do not address selection bias, omitted confounding, or the lack of external validation.

## 4. Discussion

### 4.1. Principal Findings

Three main patterns emerged from this non-probability sample. First, fear of judgment was endorsed less often by women in the decision-based group than by those in the circumstance-based group. Second, the reported route was not independently associated with changes in self-esteem/self-concept. Instead, this indicator was associated with the belief in the indispensability of fathers and perceived inequality in job access, remuneration, and labor rights. Third, pride in children’s achievements was common in both groups.

Taken together, these findings do not support a simple deficit view in which single motherhood itself explains the perceptions. They suggested that reported pathways, normative beliefs, and perceived institutional conditions may be relevant within the recruited group. Because the sample was non-representative and the data were cross-sectional, the findings do not establish population prevalence, temporal order, or causal mechanisms.

### 4.2. Behavioral Interpretation

The lower adjusted odds of fear-of-judgment endorsement in the decision-based group invite comparison with research on planned solo motherhood. However, this literature primarily examines donor-conception families and does not establish that planned entry reduces anticipated judgment (Golombok et al., 2016, 2021; Murray & Golombok, 2005; Zadeh et al., 2016; Zamora-Martínez et al., 2025). The populations were also more clearly defined by preconception planning. In contrast, the decision-based category in this study is broader, and the questionnaire did not establish when the decision was made.

Because self-determination theory treats autonomy as a basic psychological need (Ryan & Deci, 2000), future research should examine whether perceived volition is associated with anticipated judgment. The present data cannot answer this question because autonomy, agency, identity integration, and timing were not measured. Therefore, the observed association should not be interpreted as evidence that the decision to raise a child alone reduces the fear of judgment. This may reflect differences in socioeconomic resources, social support, relationship histories, or participation in the study.

The stress process model offers another interpretation of route-group differences (Pearlin et al., 1981). Paternal irresponsibility, abandonment, and family constraints may coincide with disrupted expectations, economic uncertainty, stressful events or reduced control. Comparative research similarly shows that mental health differences between single and partnered mothers are closely related to financial and social resources and unequal exposure to stressors (Cairney et al., 2003; Crosier et al., 2007; G. E. Kim et al., 2018; G. E. Kim & Kim, 2020; Rousou et al., 2016, 2019; Targosz et al., 2003). Social support may buffer these pressures (Cohen & Wills, 1985). As the study did not measure these mechanisms or their sequence, it could not identify either pathway as inherently protective or harmful. This association may reflect the circumstances surrounding entry into single motherhood or other unmeasured factors.

The association between father-indispensability beliefs and self-esteem/self-concept change endorsement is consistent with an interpretation centered on normative family beliefs. Stigma theories explain how socially preferred categories can become standards against which devalued identities are judged (Frost, 2011; Hatzenbuehler et al., 2013; Link & Phelan, 1995; Major & O’Brien, 2005; Stuber et al., 2008). Research among divorced single-parent women also links self-stigma to lower self-esteem (A. Kim et al., 2023). Intensive mothering ideology may deepen this tension by combining expectations of exhaustive care with responsibility for any perceived absence or imperfection in family life (Arendell, 2000; Hays, 1996; Henderson et al., 2016; Liss et al., 2013; Meeussen & Van Laar, 2018; Rizzo et al., 2013).

However, this interpretation remains tentative. A single response about the indispensability of fathers cannot measure internalization as a multidimensional process, and the cross-sectional design cannot establish a direction. Lower self-esteem may make the belief more salient, and both responses may reflect another unmeasured factor, or their association may partly arise because both were reported in the same questionnaire.

Perceived labor inequity was also associated with changes in self-esteem and self-concept. Several studies show that mothers may be judged as less competent or committed than otherwise comparable women, while observational research documents wage and career penalties after motherhood (Benard & Correll, 2010; Budig & England, 2001; Correll et al., 2007; Cuddy et al., 2004; Cukrowska-Torzewska & Matysiak, 2020; Deming, 2022; Gangl & Ziefle, 2009; Heilman & Okimoto, 2008). Work–family research further links incompatible role demands with psychological and occupational outcomes and identifies organizational support as a protective resource (Amstad et al., 2011; Greenhaus & Beutell, 1985; Kossek et al., 2011; Olivieri et al., 2024). For women responsible for both income and care, perceived employment inequity may coincide with material insecurity and lower social valuation.

However, the present study did not test this mechanism. The index measured perceived rather than verified employment events, employment conditions were incompletely recorded, and both the predictor and outcome came from the same self-report instrument.

Students had lower adjusted within-sample odds of fear-of-judgment endorsement than faculty members. Because this association was not hypothesized, it should be viewed as a preliminary finding. This may reflect differences in age, child characteristics, income, job security, relationship history, or exposure to workplace evaluations. Interpretation is further limited by the small faculty subgroup and the absence of information on rank and seniority. Therefore, this study could not compare junior faculty members, who may undergo frequent performance reviews, with senior faculty members.

The social meaning of single parenthood may also vary across different campuses and communities. Anticipated judgment may be less salient in places where diverse family forms are more visible and widely accepted. Campus climate, local prevalence, and perceived commonality of single parenthood were not measured. Larger studies should examine both faculty-career stages and institutional contexts before drawing conclusions about student–faculty differences.

### 4.3. Implications for Behavioral Science and Universities

The study cannot determine which university interventions would be effective because the sample is non-representative and the data are cross-sectional. Therefore, its practical implications are limited to identifying areas for further assessment. Universities could first examine anticipated judgment, caregiving constraints, and perceived employment inequity in their populations using representative institutional data and validated measures. Based on these findings, universities could formally evaluate context-specific responses, such as confidential counseling that does not frame single motherhood as a psychological deficit, stigma-reduction initiatives, transparent antidiscrimination procedures, flexible scheduling, accessible childcare, and supervisor training. These options address concerns identified in the stigma, intensive mothering, employment bias, and work–family literature (Amstad et al., 2011; Benard & Correll, 2010; Budig & England, 2001; Correll et al., 2007; Cuddy et al., 2004; Cukrowska-Torzewska & Matysiak, 2020; Deming, 2022; Frost, 2011; Gangl & Ziefle, 2009; Greenhaus & Beutell, 1985; Hatzenbuehler et al., 2013; Heilman & Okimoto, 2008; A. Kim et al., 2023; Kossek et al., 2011; Meeussen & Van Laar, 2018; Olivieri et al., 2024; Stuber et al., 2008), but the cited studies do not establish their effectiveness in Ecuadorian universities. An autonomy-supportive approach to counseling is theoretically consistent with self-determination theory (Ryan & Deci, 2000) and may also merit further evaluation. Nevertheless, the present results do not show that autonomy caused route-group differences or that any particular intervention would improve outcomes.

Universities serve as both educational institutions and employers; however, students and faculty differ in their developmental stages, income, working conditions, and exposure to evaluation. Therefore, institutional responses should be developed and assessed separately for each group. Research on student parents documents caregiving-related difficulties involving timetabling, attendance, assessment, and access to support services (Behboodi Moghadam et al., 2017; Brooks, 2012; Evans, 2025; Moreau & Kerner, 2015).

The observed association with perceived inequity in the distribution of labor identifies a topic for future institutional research rather than evidence for a particular policy. Prospective studies using validated multidimensional measures could examine whether perceived volition, anticipated stigma, family norms, social support, caregiving conditions, and verified institutional treatment are independently associated with psychosocial outcomes.

### 4.4. Strengths, Limitations, and Future Research

This study had two main strengths. First, it distinguished a decision-based response from three circumstance-based responses instead of classifying participants only by their current marital status. Second, it examined normative beliefs and perceived labor conditions while clearly separating perception-based indicators from clinical mental health outcomes.

The most important limitation of this study is the small purposive network sample. Without a sampling frame or response rate, the selection of the study cannot be quantified. Women with stronger institutional ties, greater visibility, closer connections to survey contacts, or greater willingness to discuss stigma and self-evaluation may be over-represented. Therefore, the findings apply only to the 157 respondents reached through these networks and included in the analysis. They cannot be generalized to all single mothers at the participating institutions, across Ecuadorian public universities, or in Ecuador.

The cross-sectional design also prevents temporal and causal inferences. In addition, route classification relied on a single response and may have combined heterogeneous experiences. It cannot establish preconception planning, the use of assisted reproduction, autonomy, or agency.

The measurement also limits the interpretation. Although a separate published study reported expert-based content validity evidence for the questionnaire as a whole (Zambrano Villalva et al., 2023), the present outcomes were individual response-option endorsements rather than validated multidimensional scales of stigma, fear of judgment, self-esteem, or self-concept. The earlier content validation process did not establish construct validity or reliability for these specific indicators. The responses do not measure symptom severity, clinical status, or multiple dimensions of the underlying constructs. Therefore, comparisons with studies that used validated scales are limited.

All variables were self-reported using the same online instrument. This design creates a possible common method variance and social desirability bias. Shared response tendencies may strengthen some associations, whereas a reluctance to disclose sensitive experiences may weaken others.

Residual confounding is likely because several relevant variables were not included. These include socioeconomic status and income; number, age, and other characteristics of children; childcare access; social support; prior mental health; relationship history; concurrent employment among students; detailed working conditions and job security; faculty rank; verified discrimination; and campus climate. University affiliation was recorded inconsistently, which prevented site-level adjustment, and the faculty subgroups were small.

Model diagnostics were limited to the analytical sample. The Hosmer–Lemeshow tests may have low power in a sample of this size, and the c-statistics and Brier scores were not tested in a holdout or external sample. Therefore, the regressions should not be treated as validated prediction models. The HC0 standard errors and acceptable internal diagnostics do not resolve the limitations of sampling, measurement, or confounding.

Future research should use prospective multicenter designs, probability-based or registry-supported recruitment where feasible, validated behavioral and psychosocial scales, and measures collected from more than one source or time points. Studies should also record socioeconomic, caregiving, relationship, and occupational conditions in greater detail in the future. Larger samples could distinguish specific routes into single motherhood, compare students and faculty by employment and career stage, and examine how campus context and the commonality of single parenthood shape anticipated judgment. Mediation and moderation should be tested only after the temporal order and measurement validity are established. Until then, the present results should be read as descriptive and exploratory evidence of selected perceptions in a specific recruited group, not as estimates of clinical prevalence or causal effects.

## 5. Conclusions

Among the 157 single mothers recruited through six Ecuadorian public university networks, women in the decision-based group had lower fear of judgment endorsement than women in the circumstance-based group in the adjusted secondary analyses. The reported route was not associated with changes in self-esteem or self-concept. Instead, this indicator was associated with the belief in the indispensability of fathers and perceived labor inequity.

These associations describe only the samples recruited. They do not establish population prevalence, representativeness, temporal order, or causal mechanisms, and the decision-based category is not a direct measure of agency. The findings offer hypotheses for studies with stronger sampling designs and validated measurement tools. Such evidence could help universities assess stigma, caregiving accommodations, and employment equity without treating single motherhood as a psychosocial deficit.

## Figures and Tables

**Table 1:** Participant characteristics by reported route into single motherhood.

Characteristic	Total (n = 157)	Decision-Based Route (n = 51)	Circumstance-Based Route (n = 106)	*p* Value
Age, mean (SD), years	29.99 (10.04)	30.86 (10.75)	29.57 (9.71)	0.467
Age, median (range), years	27 (18–74)	27 (18–62)	27 (18–74)	—
**University role**				0.180
Student	132 (84.1)	40 (78.4)	92 (86.8)	
Faculty	25 (15.9)	11 (21.6)	14 (13.2)	

Values are expressed as n (%) unless otherwise indicated. The *p*-value for age is from Welch’s *t*-test; the *p*-value for university role is from Pearson’s chi-square test. The current civil status is described in the text. SD: standard deviation.

**Table 2:** Emotional, social, and labor indicators by reported route into single motherhood.

Indicator	Total (N = 157)	Decision-Based Route (n = 51)	Circumstance-Based Route (n = 106)	*p* Value
Fear-of-judgment endorsement	32 (20.4)	5 (9.8)	27 (25.5)	0.022
Self-esteem/self-concept-change endorsement	62 (39.5)	16 (31.4)	46 (43.4)	0.149
Pride in child achievements	117 (74.5)	37 (72.5)	80 (75.5)	0.694
Mixed feelings	59 (37.6)	15 (29.4)	44 (41.5)	0.143
Negative social positioning	114 (72.6)	38 (74.5)	76 (71.7)	0.711
Negative religious framing	68 (43.3)	20 (39.2)	48 (45.3)	0.472
Father considered indispensable	52 (33.1)	15 (29.4)	37 (34.9)	0.493
Perceived unequal job access	52 (33.1)	12 (23.5)	40 (37.7)	0.077
Perceived unequal pay	61 (38.9)	18 (35.3)	43 (40.6)	0.526
Perceived unequal labor rights	48 (30.6)	14 (27.5)	34 (32.1)	0.556
Workplace treatment perceived to change	135 (86.0)	44 (86.3)	91 (85.8)	0.943
Deficient work performance endorsed	91 (58.0)	30 (58.8)	61 (57.5)	0.879
Labor inequity index, mean (SD)	1.03 (1.12)	0.86 (1.04)	1.10 (1.15)	0.192

Values are expressed as n (%) unless otherwise indicated. The outcomes represent endorsements of survey response options rather than clinical diagnoses. Pearson’s chi-square test was used for binary indicators, and Welch’s *t*-test was used for the labor inequity index. The labor inequity index ranged from 0 to 3 and summed the perceived inequality in job access, pay, and labor rights. SD: standard deviation.

**Table 3:** Secondary exploratory logistic regression models for perception-based indicators.

Outcome and Predictor	aOR	95% CI	*p* Value
**Fear-of-judgment endorsement**			
Decision-based route (vs. circumstance-based)	0.25	0.08–0.77	0.016
Age, per 5-year increase	0.90	0.72–1.12	0.355
Student role	0.18	0.06–0.55	0.003
**Self-esteem/self-concept-change endorsement**			
Decision-based route (vs. circumstance-based)	0.64	0.30–1.36	0.247
Age, per 5-year increase	0.83	0.67–1.01	0.066
Student role	0.38	0.13–1.17	0.091
Father considered indispensable	2.91	1.41–5.99	0.004
Labor inequity index, per 1-point increase	1.57	1.15–2.13	0.004

aOR, adjusted odds ratio; CI, confidence interval. The circumstance-based group and faculty roles were the reference categories. The models used HC0 robust standard errors. The fear-of-judgment model included route group, age, and role (32 events, 10.7 events per predictor). The self-esteem/self-concept-change model additionally included the father-indispensability belief and the labor inequity index (62 events; 12.4 events per predictor). Estimates are secondary within-sample adjusted summaries; robust standard errors and favorable internal diagnostics do not address non-probability sampling, selection bias, omitted confounding, or external validity. VIFs, likelihood ratio tests, Hosmer–Lemeshow tests, apparent C-statistics, Brier scores, and influence diagnostics are reported in Section 3.3.

## Data Availability

The data supporting the findings of this study are available from the corresponding author upon reasonable request, subject to institutional and ethical requirements for confidentiality. The data are not publicly available due to privacy and ethical reasons.
